# Sexual Function of Women with and without Pregnancy-Related Pelvic Girdle Pain and its Relationship with Physical Activity, Kinesiophobia and Body Image: A Cross-Sectional Comparative Study

**DOI:** 10.1007/s43032-024-01644-2

**Published:** 2024-07-09

**Authors:** Semiha Yenişehir, İlkim Çıtak Karakaya, Gürkan Özbey

**Affiliations:** 1 Faculty of Health Sciences, Department of Physiotherapy and Rehabilitation, Muş Alparslan University, 49100 Muş, Turkey; 2 Faculty of Health Sciences, Department of Physiotherapy and Rehabilitation, Muğla Sıtkı Koçman University, 48000 Muğla, Turkey; 3 Department of Obstetrics and Gynecology, Private Anadolu Hospital, Elazığ, Turkey

**Keywords:** Activity limitation, Body image, Kinesiophobia, Posterior pelvic pain, Sexual dysfunction

## Abstract

The aim of this study was to compare the sexual function of women with and without pregnancy-related PGP, and to investigate its relationship with physical activity (PA), kinesiophobia, and body image (BI). Demographic characteristics, sexual function (Pregnancy Sexual Response Inventory), PA (Pregnancy Physical Activity Questionnaire), kinesiophobia (Tampa Kinesiophobia Scale), and BI (Body Image in Pregnancy Scale) of 125 pregnant women were recorded. In the PGP group (*n* = 46), visual analogue scales were used to assess the pain intensity during resting and sexual activity, and Pelvic Girdle Questionnaire was used to evaluate the activity limitation. Although total sexual function and BI scores of the groups were similar (p > 0.05), dyspareunia during pregnancy and level of kinesiophobia were higher, and energy expenditure during moderate-intensity PA was lower in pregnant women with PGP (*p* < 0.05). The PGP group had moderate activity limitation and reported increased PGP intensity during sexual activities (*p* < 0.001). PA level was significantly correlated with sexual desire (r = 0.180), and overall sexual function was correlated with kinesiophobia (r = -0.344) and BI (r = -0.199) during pregnancy (*p* < 0.05). These findings suggest that pregnant women with PGP are more vulnerable to sexual dysfunctions, and there is a need to develop biopsychosocial framework-oriented management strategies which aim to improve PA level and to eliminate psychological factors such as kinesiophobia and negative BI.

**Clinical Trial Registration**: NCT05990361

## Introduction

Sexual function is defined as how the body reacts in the different stages (excitement, plateau, orgasm, and resolution) of the sexual response cycle [1], and is characterized by not only absence of difficulty moving through these stages but also having subjective satisfaction with the frequency and outcome of individual and partnered sexual behavior [2].

Women's sexual functions and behaviors may be affected by pregnancy-related physical, physiological, emotional and body image changes, and attitude toward sex during pregnancy, health-related quality of life (QoL) and adverse symptoms during sex are the determinants of sexual function during this period [3]. Sexual dysfunction, which has an enormous effect on QoL and psychological wellbeing is a very frequent problem with a pooled global prevalence of 70% in pregnant women [3]. Studies have shown that sexual function worsens during pregnancy, is not fully recovered by six months after birth, and pregnant women with lumbar or pelvic pain are more likely to be dissatisfied with their sex lives [4–6].

Pelvic girdle pain (PGP) is a frequent problem with an approximately 20%-point prevalence in pregnant women and has a significant impact on various aspects of life, including limitations in physical, occupational, social, and recreational activities, as well as problems in personal relationships and marital life, as in sexual dysfunction [7]. Pregnant women with PGP also have higher levels of kinesiophobia than their pain-free counterparts, which prevents them from engaging in physical activities due to the thought that movement will cause re-injury and increase pain [8].

Physical demands of sexual intercourse overlap with demands of the physical activity (energy expenditure, perceived exertion, heart rate, blood pressure, and body kinematics changes, etc.), so can be considered as a form of physical activity with varying intensity depending on factors such as sex, health status, intercourse position, and activity duration [9].

Although the literature includes some knowledge about the effects of pregnancy-related PGP on physical activities and daily living, as well as the effects of persisting PGP after childbirth on sexuality, sexual function of pregnant women with PGP is neglected and remains under researched despite its obvious effects on emotional and physical health [10–12].

Therefore, the purpose of this study is to examine the sexual function of pregnant women with PGP in comparison with their pain-free counterparts, and to find out if it is correlated with their body image, physical activity and kinesiophobia levels.

## Methods

This cross-sectional observational study was conducted at Obstetrics and Gynecology Department of Muş State Hospital between June and July 2023 following the ethical approval obtained from the Scientific Research Ethical Board of the Muş Alparslan University (date:18/04/2023, protocol number:4–35). The Clinical Trials registration number is NCT05990361.

In accordance with the Helsinki Declaration of Ethics Principles, written informed consent was obtained from the participants.

## Subjects

In this study, the second and third trimester pregnant women aged between 18–40 years and literate in Turkish were included. Multiple pregnancy; pregnancy complications (preeclampsia, pregnancy-induced hypertension, diabetes, etc.); gynecological/urological problems that may mimic PGP; neurological, orthopedic, cardiopulmonary problems that affect sexual function and physical activity (multiple sclerosis, spinal cord injury, hip dislocation, heart failure, chronic obstructive pulmonary disease, etc.); health conditions requiring restriction of sexual activity; visual/auditory/cognitive problems that may prevent participation; and low back and/or pelvic pain unrelated with PGP were the exclusion criteria for the study.

In order to classify the participants who had low back and/or pelvic pain as with or without PGP, active straight leg raise (ASLR) [13], posterior pelvic pain provocation (P4) [14], long dorsal sacroiliac ligament palpation (LDSLP) [15], pelvic compression [14], pelvic distraction [14], Patrick-Faber (PF) [14], and Gaenslen (GT) [15] tests were used as recommended in the European guidelines for the diagnosis and treatment of PGP [15]. The subjects who had at least one positive result from ASLR and P4 tests; and at least two positive results from the pelvic compression, pelvic distraction, PF, GT and LDSLP tests were classified as the PGP group [16].

In the PGP group, the intensity of pelvic pain during resting and sexual activity was assessed with 0–10 cm Visual Analogue Scales (VASs), where the zero point indicated “no pain”, and the 10 cm point indicated “unbearable pain”. In clinical practice, VAS scores are categorized as between 0 and 3 as mild pain, between 3 and 6 as moderate pain, and > 7 as severe pain [17].

Participants' physical (age, height, body weight and body mass index), sociodemographic (education, marital and occupational status), and obstetrical characteristics (gestational week, number of pregnancies and births) were recorded.

The sexual function of all participants was assessed by the Pregnancy Sexual Response Inventory (PSRI) which consists of 38 items and two sections [18, 19]. The first section includes 12 items about demographic characteristics of pregnant women. The second section includes questions regarding the sexual function and is divided into nine subscales: (1) the frequency of sexual activity, (2) sexual desire, (3) sexual satisfaction, (4) arousal, (5) orgasm, (6) dyspareunia, (7) sexual difficulty and dysfunction, (8) beginning of sexual intercourse, and (9) the opinion of the pregnant woman’s partner on the sexual response. For each subscale, there are two periods of “before pregnancy” (11 questions) and “during pregnancy” (15 questions). The total score ranges from 0 to 100 points, and the scores can be categorized as “rubbish” (0–25 points), “bad” (25–50 points), “good” (50–75 points), and “excellent” (75–100 points) [18, 19].

Additionally, to assess the disability/activity limitation and symptom severity, the Pelvic Girdle Questionnaire (PGQ) which is a reliable and valid 25-itemed measure including activity (20 items) and symptom (5 items) subscales was used [20, 21]. Each item was rated on a 4-point scale ranging from “no problem at all (score 0)”, “to a large extent (score 3)”, and the maximum possible score was 75 (60 for the activity and 15 for the symptom subscales) [18, 19]. The values 0–28 were interpreted as low, 28–62 as moderate, and > 62 as high disability [22]. Also, the total PGQ scores were summarized, divided by 75 and multiplied with 100 to get the percentage values, in order to be comparable with the findings of other relevant studies which reported the percentage values [20, 21].

The Pregnancy Physical Activity Questionnaire (PPAQ) which consists of 32 activities were classified by type (household/caregiving, occupational, and sports/exercise). The average MET-hours per week spent in each activity type was calculated. The average daily energy expenditure was determined by multiplying the number of hours spent for each activity by the intensity of the activity [23, 24].

The kinesiophobia level of the participants was assessed by the Tampa Kinesiophobia Scale (TKS), which includes 17 items, each rated on a 4-point Likert scale ranging from 1 (strongly disagree) to 4 (strongly agree). Four items (4, 8, 12 and 16) are reverse scored statements [25, 26]. Total scores range from 17 to 68, with scores above 37 indicate a “high degree” of fear of movement [26].

The body image (perceptions of physical and mental changes in the body) of the groups was assessed with the Body Image Pregnancy Scale (BIPS). The Turkish version of BIPS consists of 34 items and seven subscales. These subscales include: preoccupation with physical appearance (items 1–4, 33, 34), dissatisfaction with strength-related aspects of one’s body (items 13–19), dissatisfaction with complexion (items 20–23), sexual attractiveness (items 5–7), prioritization of appearance over function (items 8–12), appearance-related behavioral avoidance (items 30–32), and dissatisfaction with body parts (items 24–29). The 8-12th items are reverse scored, and the total score obtained from the scale reflects the body image perception, where the higher scores indicate more negative perceptions [27, 28].

## Statistical Analysis

Sample size was calculated using the G-Power analysis (Mac version 3.1.9.3). Since both between-group comparisons and the correlation analysis would be required for the study, two different calculations were done. For between-group comparisons, “t-test family: difference between two independent groups” and the effect size: 0.5, alpha level: 0.05, power: 85% was selected. The calculated sample size was 118 participants. For the correlation analysis “bivariate normal model”, effect size: 0.3, alpha level: 0.05, power: 95% was selected, and the sample size was calculated as 115. Considering both calculations, it was decided to include at least 118 participants for the study.

The Statistical Package for the Social Sciences (version 25.0 for MAC) was used for data analysis. Quantitative variables were described as mean ± standard deviation (SD), and qualitative variables as number (n) and percentage (%). The Kolmogorov–Smirnov test was used to determine whether data were normally distributed or not. Chi-square test was used for inter-group comparisons of the categorical data. Due to the presence of data which does not comply with normal distribution, the Mann–Whitney u test was used to compare the physical characteristics, as well as the PSRI, BIPS, TKS and PPAQ scores between groups. Wilcoxon signed rank test was used for comparing sexual function between pre-pregnancy and pregnancy period. The correlations between the PSRI, BIPS, TKS, PPAQ, and PGQ scores were investigated using the Spearman correlation analysis. Correlation coefficients were interpreted as poor (r:0.10–0.30), moderate (r:0.30–0.70), or strong (r:0.70–0.100) [29]. Multiple linear regression analysis was used to determine to what extent kinesiophobia (TKS), physical activity (PPAQ) and body image (BIPS) as independent variables were effective on sexual function (PSRI) (dependent variable). Multivariate logistic regression analysis was performed to determine the factors independently associated with PGP (dependent variable). Statistical significance was set at 95% confidence interval (CI) and *p* < 0.05.

## Results

**Fig. 1:**
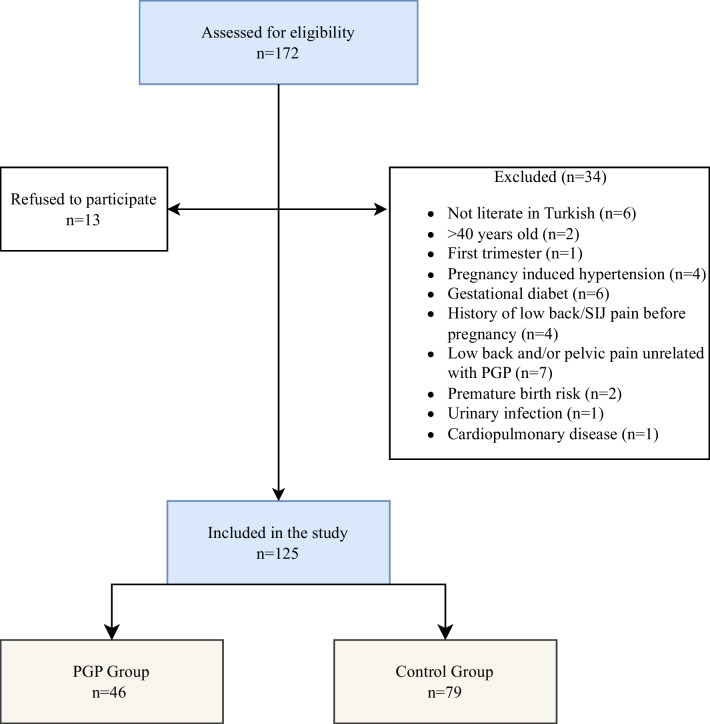
Flow chart of subjects

As shown in the flow diagram, 172 pregnant women were interviewed and 125 pregnant women participated in the study after signing the informed consent forms (Fig. 1). Among the participants, 46 (38.7%) were determined to have pregnancy-related PGP according to the criteria as mentioned in the method section.

The mean age (year) of all participants was 26.5 ± 5.12 years. All of them were married, 88% were housewives, and most of them had a low education level (Table 1). The physical and sociodemographic characteristics of the groups were similar (*p* > 0.05) (Table 1). Gravida and parity of the PGP group were higher than the control group (*p* < 0.05) (Table 2). Nearly three quarters of participants (56 women with and 35 without PGP) were in the third trimester, and the groups were similar in regard to the pregnancy stage (x^2^ = 0.397, *p* = 0.529).

**Table 1:** Physical and sociodemographic characteristics

	PGP Group *n* = 46 Mean ± SD	Control group *n* = 79 Mean ± SD	u	*p*
Physical characteristics				
Age (year)	27.37 ± 4.86	25.87 ± 4.94	1463.50	0.070
Height (m)	1.62 ± 0.05	1.63 ± 0.04	1589.50	0.243
Pre-pregnancy weight (kg)	64.07 ± 12.56	63.52 ± 10.69	1795.00	0.910
Pregnancy weight (kg)	72.61 ± 12.52	71.37 ± 10.35	1795.50	0.912
Pre-pregnancy BMI (kg/m^2^)	24.36 ± 4.16	23.85 ± 3.68	1705.00	0.566
Pregnancy BMI (kg/m^2^)	27.51 ± 4.06	26.80 ± 3.50	1658.50	0.417
Sociodemographic characteristics				
	**n (%)**	**n (%)**	**x**^**2**^	**p**
Education				
Primary school	26 (56.5)	54 (68.4)	6.197	0.102
Secondary school	10 (21.7)	8 (10.1)		
High school	2 (4.3)	9 (11.4)		
University	8 (17.4)	8 (10.1)		
Occupation				
Housewife	40 (87)	70 (88.86)	1.699	0.889
Teacher	4 (8.7)	5 (6.3)		
Office worker	1 (2.2)	3 (3.9)		
Other	1 (2.2)	1 (1.3)		

*PGP* pelvic girdle pain; *BMI* body mass index; *SD* standard deviation; *u* Mann–whitney-u test value; *x*^*2*^ chi-square value; *p* statistical significance value

**Table 2:** Obstetrical history

	PGP Group *n* = 46 Mean ± SD [Median]	Control Group *n* = 79 Mean ± SD [Median]	u	*p*
Gravida	3.33 ± 1.85 [3.00]	2.56 ± 1.75 [2.00]	1362.500	0.017*
Abortus	0.37 ± 0.80 [0.00]	0.25 ± 0.72 [0.00]	1677.500	0.272
D&C	0.20 ± 0.45 [0.00]	0.09 ± 0.33 [0.00]	1640.000	0.097
Parity	1.78 ± 1.49 [2.00]	1.09 ± 1.40 [1.00]	1302.500	0.006*
Vaginal birth	1.39 ± 1.51 [1.00]	0.92 ± 1.41 [0.00]	1472.500	0.053
Cesarean section	0.39 ± 0.68 [0.00]	0.23 ± 0.58 [0.00]	1598.500	0.114

*PGP* pelvic girdle pain; *D&C* dilation and curettage; *SD* standard deviation; *u* Mann–Whitney-u test value; *: *p* < 0.05

In the PGP group, the intensity of pain was moderate at rest, and was higher during sexual activity (5.09 ± 0.65 cm and 5.60 ± 0.59 cm on 0–10 cm VASs, respectively; t = -6.122, *p* < 0.001). According to the total mean PGQ scores (46.02 ± 7.11 points and 61.36 ± 9.48%), the PGP group had a moderate level of disability/activity limitation and symptom severity. The mean disability/activity limitation score was 38.09 ± 6.05 points (50.99 ± 8.03%), and the mean symptom severity score was 7.93 ± 2.70 points (10.58 ± 3.60%).

Within-group comparisons have shown the sexual function of women both with and without PGP decreased during pregnancy (z = -5.895 and -7.722, respectively, and *p* =  < 0.001), and while both groups had a “good” (50–75 points) sexual function before, it was “rubbish” (0–25 points) during pregnancy (Table 3). Although pre-pregnancy mean total and subscale scores of the PSRI was similar between the groups (*p* > 0.05), the PGP group had a lower dyspareunia subscale score during pregnancy, indicating that more women in the PGP group have reported dyspareunia than the control group in this period (*p* < 0.05) (Table 3).

**Table 3:** Comparison of the sexual function (PSRI score), physical activity (PPAQ score) and kinesiophobia (TKS score) levels, and body image (BIPS score) of the groups

		PGP Group *n* = 46 Mean ± SD	Control Group *n* = 79 Mean ± SD	u	*p*
PSRI-before pregnancy total and subscales scores					
Total score		59.13 ± 9.43	56.74 ± 9.39	1611.00	0.288
Frequency		54.35 ± 14.24	58.86 ± 22.30	1649.00	0.193
Desire		20.65 ± 29.01	20.89 ± 24.82	1762.50	0.744
Arousal		51.09 ± 7.37	50.00 ± 0.00	1777.50	0.190
Orgasm		51.09 ± 30.71	42.41 ± 30.05	1560.00	0.127
Satisfaction		62.50 ± 15.59	57.28 ± 16.09	1558.00	0.139
Dyspareunia		78.26 ± 40.35	73.42 ± 42.24	1708.500	0.479
Intercourse start		51.09 ± 12.86	50.00 ± 8.01	1778.00	0.556
Female difficulties		91.30 ± 19.16	94.30 ± 15.99	1708.00	0.556
Male sexual satisfaction		52.17 ± 14.74	51.27 ± 11.25	1784.00	0.690
Male sexual difficulties		73.91 ± 41.82	68.35 ± 44.71	1712.00	0.518
PSRI-during pregnancy total and subscales scores					
Total score		22.49 ± 12.76	23.35 ± 10.42	1.665.50	0.438
Frequency		30.42 ± 11.81	32.90 ± 9.97	1.615.50	0.251
Desire		7.61 ± 12.77	11.39 ± 13.15	1536.50	0.090
Arousal		33.70 ± 23.70	34.81 ± 23.14	1776.50	0.796
Orgasm		16.30 ± 23.70	16.46 ± 23.64	1811.50	0.972
Satisfaction		21.74 ± 20.82	18.35 ± 20.69	1646.50	0.347
Dyspareunia		21.74 ± 41.70	39.87 ± 48.95	1481.00	0.036*
Intercourse start		42.61 ± 18.31	41.90 ± 18.75	1781.00	0.780
Female difficulties		19.57 ± 40.11	11.39 ± 31.97	1708.00	0.347
Male sexual satisfaction		13.04 ± 34.05	6.32 ± 24.50	1658.50	0.346
Male sexual difficulties		13.04 ± 34.05	6.33 ± 24.50	1695.00	0.203
PPAQ-total score and subscales (MET.h/wk)					
Total score		93.28 ± 41.02	101.72 ± 33.09	1433.00	0.049*
Intensity	Sedentary (< 1.5 METs)	20.52 ± 17.50	19.73 ± 20.50	1607.00	0.281
	Light (1.5–3.0 METs)	55.66 ± 27.92	58.41 ± 18.49	1453.00	0.062
	Moderate (3.0–6.0 METs)	15.99 ± 14.08	24.16 ± 17.95	1292.00	0.007*
	Vigorous (> 6 METs)	1.10 ± 2.81	0.85 ± 2.21	1786.00	0.795
Type	Household/caregiving	39.06 ± 34.89	37.92 ± 31.79	1814.50	0.990
	Occupational activity	6.35 ± 23.21	7.07 ± 25.51	1815.00	0.983
	Sports/exercise	6.76 ± 6.64	6.72 ± 10.00	1618.50	0.303
TKS		37.87 ± 4.01	35.82 ± 4.39	1304.00	0.008*
BIPS-Total score		92.74 ± 9.01	90.81 ± 10.23	1620.00	0.313

*PSRI* Pregnancy Sexual Response Inventory; *BIPS* Body Image in Pregnancy Scale; *PPAQ* Pregnancy Physical Activity Questionnaire; *TKS* Tampa Kinesiophobia Scale; *SD* Standard deviation; *u* Mann–Whitney test value; *: *p* < 0.05

While the PGP group had a similar energy expenditure with the control group at sedentary, light and vigorous physical activity intensities, as well as during the household/caregiving, occupational and sports/exercise activities (*p* > 0.05), it was found that they were spending less time during moderate-intensity physical activities and their overall physical activity level was lower than the control group (*p* < 0.05) (Table 3).

According to the TKS scores, 63% of the PGP group and 40.5% of the control group had higher kinesiophobia (> 37 points), and the PGP group had more fear of movement than the control group (*p* < 0.05) (Table 3).

There was no significant difference between the mean total BIPS scores of the groups, indicating that the body image perception of the groups was similar (*p* > 0.05) (Table 3).

The total PSRI scores showed a moderate negative correlation with the TKS score (r = -0.344) and a poor negative correlation with the total BIPS (r = -0.199) scores, indicating that sexual function gets worse as the fear of movement and negative perception of body image increase (*p* < 0.05) (Table 4).

**Table 4:** Correlations of sexual function** (**PSRI-during pregnancy score) with physical activity (PPAQ-total score), kinesiophobia (TKS score), and body image (BIPS total score) (*n* = 125)

	PPAQ-Total		BIPS-Total		TKS	
	r	*p*	r	*p*	r	*p*
PSRI-Total	0.153	0.089	-0.199	0.026*	-0.344	< 0.001*
Frequency	0.019	0.832	-0.101	0.260	-0.190	0.034*
Desire	0.180	0.045*	-0.191	0.033*	-0.272	0.002*
Arousal	0.048	0.592	0.020	0.824	-0.053	0.558
Orgasm	-0.056	0.534	0.134	0.137	0.026	0.771
Satisfaction	0.121	0.179	-0.153	0.088	-0.180	0.045*
Dyspareunia	0.155	0.085	-0.128	0.156	-0.162	0.070
Intercourse start	0.059	0.512	0.170	0.058	-0.104	0.251
Female difficulties	0.009	0.920	-0.146	0.105	-0.169	0.059
Male sexual satisfaction	0.031	0.731	-0.349	< 0.001*	-0.359	< 0.001*
Male sexual difficulties	-0.008	0.928	-0.073	0.420	-0.009	0.920

*PSRI* Pregnancy Sexual Response Inventory; *PPAQ* Pregnancy Physical Activity Questionnaire; *TKS* Tampa Kinesiophobia Scale; *BIPS* Body Image in Pregnancy Scale; *r* Spearman’s correlation coefficient; *: *p* < 0.05

Although no significant correlation was found between the total scores of sexual function during pregnancy and physical activity (*p* > 0.05), according to the PSRI-subscale correlations, the level of physical activity was poorly correlated with sexual desire which was also poorly correlated with the body image and fear of movement (*p* < 0.05). The frequency of sexual activity and satisfaction with the sexual life were other parameters of sexual function poorly correlated with the level of kinesiophobia. According to the perceptions of pregnant women, sexual satisfaction of their partner was moderately correlated with their body image and fear of movement (*p* < 0.05) (Table 4). These correlations point out that sexual desire increases as the level of physical activity and positive body image increase, and as the fear of movement decreases; the frequency of sexual activity and satisfaction decrease as the fear of movement increases; and the male sexual satisfaction increases as the body image gets positive and fear of movement decreases.

In the multiple regression model, sexual function was taken as the dependent variable, while physical activity, body image and kinesiophobia were taken as independent variables. As a result of multiple regression analysis, the model was statistically significant (F = 5.493, *p* = 0.001), and showed a good fit (VIF < 1.2 and tolerance > 0.86). In the model, 12% of the variance in sexual function was explained by the independent variables in all pregnant women (R = 0.346, R2 = 0.120). Kinesiophobia was found to have a significant effect on sexual function (*p* = 0.001), where physical activity and body image had no significant effect on sexual function (*p* > 0.05). Multivariate logistic regression analysis showed that kinesiophobia was the only independent variable associated with PGP (OR = 1.126, *p* = 0.020, 95%CI for OR = 1.019–1.243).

## Discussion

The main findings of this study show that the sexual function of women gets worse during pregnancy, and although there is no difference of general sexual function between pregnant women with and without PGP, the PGP group experiences more dyspareunia than the pain-free counterparts, and their intensity of PGP increases during the sexual activity.

Many previous trials which investigated sexual function of pregnant women have reported a decrease in sexual function during pregnancy even though the samples were women from different countries/cultures [22, 30, 31]. The related findings of this study are in parallel with these trials and also contribute to the literature by presenting that pregnant women both with and without PGP experience a decline in their sexual function during pregnancy.

In the literature, there is a limited number of studies which investigated the effect of musculoskeletal pain of the obstetric population on their sexual function [5]. Mogren (2006) has conducted a study on women who had low back pain (LBP) or PGP during pregnancy and gave birth within 24 h, and retrospectively questioned about their sexual life during pregnancy [5]. Upon logistic regression analysis, she concluded that LBP and PGP demonstrated a negative impact on perceived health and sexual life during pregnancy. However, this was not a comparative study between pregnant women with and without pain. Another study conducted on postnatal women showed that pain during sexual intercourse was more frequent in women with persistent PGP and these women often avoided sexual intercourse when compared with healthy counterparts [11]. The findings of the current study, although presenting no difference between the groups in aspect of general sexual function scores highlights that pregnant women with PGP experience more complaints of dyspareunia, and support the results of previous trials, which point out that PGP and/or LBP can lead to increased dyspareunia complaints in the obstetrical population. As well known, the etiology of dyspareunia is multifactorial and include cognitive-affective (such as sexual abuse, fear, and pain catastrophizing), biomedical (such as hormonal changes, and pelvic floor muscle disorders), cultural (such as attitude toward sexuality), and relational factors (such as the sexual partner’s reactions) [32]. Among these, hormonal changes and pelvic floor muscle disorders are common factors associated with the etiology of pregnancy-related PGP [15]. The possible mechanism of interaction between the PGP and dyspareunia can be explained through the findings of previous studies which showed that women with musculoskeletal pain including PGP were more likely to have increased pelvic floor muscle activity and levator ani tenderness which could lead to pain during vaginal intercourse [11], as well as the findings indicating that pain with intercourse is a potential indicator of enhanced supraspinal involvement in nociceptive processing and is significantly associated with evoked pain perception and cognitive aspects of pain in women with pelvic pain [33].

In this study, the PGP group reported a significant increase in PGP intensity during sexual activity, which can be explained by biomechanical mechanisms. As known, ligamentous laxity compromises pelvic stability, and this frequently contributes to sacroiliac joint dysfunction or pubic symphysis separation, which significantly limits pelvic and extremity movements typical to sexual activity including lumbar flexion, pelvic tilting and rocking, hip flexion, abduction and external rotation [9, 34]. Sidorkewicz and McGill (2015) have shown that, regardless of the coital position, female coital movement was cyclic and predominantly in the sagittal plane of motion, and the female LBP patients had experienced difficulty in finding a comfortable position and difficulty with pelvic movements during coitus [35]. The findings of both the current and the previous studies indicate the need for further studies on the biomechanical analysis of female lumbar spine and pelvic kinematics during coitus to provide empirical data that will strengthen coital motion and posture adjustment recommendations for pregnant and nonpregnant populations with LBP and PGP.

 As a finding consistent with the results of the previous relevant studies, the present study showed that the PGP group had a lower physical activity level, was less engaged in moderate-intensity physical activities and had more fear of movement than the control group [8, 36, 37].

Considering that sexual intercourse is a type of moderate-intensity physical activity that can lead to energy expenditure of ~ 6 METs [9], overall sexual function, and particularly scores on the desire and frequency subscales of the PGP group, were expected to be significantly lower than those of the control group. However, statistical analysis of these PSRI data showed no significant difference of overall sexual function between groups. This situation is thought to be probably due to the fact that sexual function has already decreased due to pregnancy and was “rubbish” not only in the PGP group but also in the control group.

Correlational analysis results indicated that kinesiophobia was the common factor significantly related with the desire, frequency, and partners’ satisfaction components as well as the overall sexual function. Considering these relationships and the finding that the PGP group had more fear of movement than the control group, it can be concluded that kinesiophobia is a very important psychological factor that disrupts the sexual life of not only pregnant women with PGP but also their partners. Although the literature is limited in knowledge regarding the role of kinesiophobia on sexual functioning of people with LBP or PGP, the results of the current study support the findings of Ferrari et al. (2019), that investigated the relevance and characteristics of sexual disability due to LBP, and showed by regression analysis that sexual disability was related to activity avoidance component of the fear of movement as measured with TSK [38].

In this study, it was observed that pregnant women with and without PGP had similar body image perceptions. This finding is inconsistent with the findings of studies by Wand et al. (2017) and Goossens et al. (2021), indicating that pregnant women with lumbopelvic pain exhibited a significantly more disturbed body perception compared to pain-free women [39, 40]. This discrepancy may be due to the fact that while both of these studies were conducted on women in the last trimester or month of pregnancy, the current study also included women in the second trimester, when the change in body image had not yet reached its maximum. As a supporting evidence to this interpretation, Linde et al. (2022) have stated that, the body image has a multidimensional nature that encompasses a person's self-perceptions, cognitions, emotional states, and behaviors related to physical characteristics, and there are different courses for different components of body image throughout the pregnancy period [41]. Another explanation to this conflict may be the different outcome measures used in these studies to assess body image perception. While both studies by Wand et al. (2017) and Goossens et al. (2021) have used Fremantle Back Awareness Questionnaire (FreBAQ) which assesses body perception specifically at the lumbopelvic region, BIPS which is a multidimensional measure designed to cover key features of body image, including body dissatisfaction, importance and ideals of body image, pregnancy-related body changes, functioning of the pregnant body, sexual attractiveness, and appearance-related behaviors was used in the current study [42]. Therefore, in order to reach a definitive conclusion about the effect of PGP or LBP on pregnant women's body image perceptions, it is thought that future studies should compare the body image of women with and without pain in groups specific to different pregnancy stages, and body image assessment should be made using pregnancy-specific, multidimensional measures.

In this study, body image was found to be correlated with the overall sexual function as well as its desire and male satisfaction components. Similarly, in another study conducted in the same country, Gümüşay et al. (2021) showed that pregnant women's positive body image perception was associated with the positive sexual function of both women and their partners [43]. Additionally, Paul et al. (2008) pointed out an association between body image and sexual function, mainly in the first trimester [4]. However, based on data of Iranian women in their 2nd and 3rd trimesters of pregnancy, Senobari et al. (2019) have stated that there was no relationship between sexual function and body image [44]. The fact that studies were conducted on individuals from different cultures using different sexual function and/or body image scales may explain the contradictory findings on this subject.

## Strengths and Limitations

The main strengths of the present study are that, the PGP diagnosis was based on the recommendations of an international guideline and the effect of PGP on sexual function of pregnant women was analyzed by comparison of the two groups which had similar physical and sociodemographic characteristics; the outcome measures used were reliable and valid tools for the pregnant population, and were multidimensional in general; and the correlational analysis included not only the total but also the subscale scores of the PSRI to comprehensively investigate the relations of its different components.

The present study also has some limitations. Firstly, all the outcome measures used in this study are patient-reported, and no objective tool has been used. Additionally, the PSRI before pregnancy have recall bias which may challenge the validity of responses. Secondly, level of physical activity was not measured using a direct method but was evaluated through a self-report questionnaire that provided only estimated values of the time spent for activities and total energy expenditure. Thirdly, there is a lack of a pre-pregnancy physical activity level inquiry of the sample that could have an important role in their current sexual function, kinesiophobia and body image. Finally, the only investigated psychological factor was kinesiophobia in this study although there are some others such as pain vigilance, catastrophizing and fear-avoidance behaviors, which can be related with the sexual function of pregnant women especially for the ones with PGP.

## Conclusion

Understanding sexual dysfunction in pregnant women with and without PGP and its relationship with PA, body image, and kinesiophobia may help developing targeted management strategies in terms of women's health approaches.

This study emphasizes that PGP is a problem that reduces the level of PA in pregnant women and increases kinesiophobia and dyspareunia complaints, as well as it points out that low PA level, negative body image and especially kinesiophobia, may be associated with sexual dysfunctions related with desire, frequency and satisfaction components in pregnant women.

These findings highlight that sexual dysfunction in pregnant women with PGP is a problem that should not be ignored, and reveal the need to develop biopsychosocial framework-oriented management strategies to increase their PA levels, reduce negative psychological factors such as kinesiophobia, and improve sexual function.

## Data Availability

Not applicable.
